# Intravenous immunoglobulin G therapy for neonatal hyperbilirubinemia

**DOI:** 10.1038/s41390-023-02712-0

**Published:** 2023-07-25

**Authors:** Saisujani Rasiah, Thivia Jegathesan, Douglas M. Campbell, Prakeshkumar S. Shah, Michael D. Sgro

**Affiliations:** 1 Department of Pediatrics, St. Michael’s Hospital, Toronto, ON Canada; 2 Keenan Research Centre of the Li Ka Shing Knowledge Institute, St Michael’s Hospital, Toronto, ON Canada; 3 Department of Pediatrics, University of Toronto, Toronto, ON Canada; 4 Maternal-Infant Care Research Centre, Mount Sinai Hospital, Toronto, ON Canada; 5 Department of Pediatrics, Mount Sinai Hospital, Toronto, ON Canada; 6 Institute of Medical Science, University of Toronto, Toronto, ON Canada

## Abstract

**Background:**

Neonatal hyperbilirubinemia (NHb) results from increased total serum bilirubin and is a common reason for admission and readmission amongst newborn infants born in North America. The use of intravenous immunoglobulin (IVIG) therapy for treating NHb has been widely debated, and the current incidence of NHb and its therapies remain unknown.

**Methods:**

Using national and provincial databases, a population-based retrospective cohort study of infants born in Ontario from April 2014 to March 2018 was conducted.

**Results:**

Of the 533,084 infants born in Ontario at ≥35 weeks gestation, 29,756 (5.6%) presented with NHb. Among these infants, 80.1–88.2% received phototherapy, 1.1–2.0% received IVIG therapy and 0.1–0.2% received exchange transfusion (ET) over the study period. Although phototherapy was administered (83.0%) for NHb, its use decreased from 2014 to 2018 (88.2–80.1%) (*P* < 0.01). Similarly, the incidence of IVIG therapy increased from 71 to 156 infants (1.1–2.0%) (*P* < 0.01) and a small change in the incidence of ET (0.2–0.1%) was noted.

**Conclusion:**

IVIG therapy is increasingly being used in Ontario despite limited studies evaluating its use. The results of this study could inform treatment and management protocols for NHb.

**Impacts:**

- Clinically significant neonatal hyperbilirubinemia still occurs in Ontario, with an increasing number of infants receiving Intravenous Immunoglobulin G (IVIG) therapy.
- IVIG continues to be used at increasing rates despite inconclusive evidence to recommend its use.
- This study highlights the necessity of a future prospective study to better determine the effectiveness of IVIG use in treating neonatal hyperbilirubinemia, especially given the recent shortage in IVIG supply in Ontario.
- The results of this study could inform treatment and management protocols for neonatal hyperbilirubinemia.

## Introduction

Neonatal hyperbilirubinemia (NHb) is a common reason for admission and readmission amongst infants born in North America.^1–3^ Acute and chronic bilirubin encephalopathy (ABE and CBE) are serious irreversible neurological complications that could appear in infants who develop severe hyperbilirubinemia.^4^ Visible jaundice in newborn infants is secondary to a serum-elevated bilirubin and occurs in 60–80% of newborn infants in the first week of life.^5^

At the turn of the century, it was estimated that 1 in 2480 live births presented with severe hyperbilirubinemia (bilirubin level ≥425 μmol/L) in Canada.^6^ After the Canadian Pediatric Society (CPS) and American Academy of Pediatrics introduced guidelines for management and treatment of NHb by 2007, the incidence decreased significantly to approximately 1 in 8352 live births.^4,7,8^ Current standard guidelines apply to infants born at 35 weeks or greater gestational age (GA). Clinically significant hyperbilirubinemia only occurs in a small percentage (<10%) of infants (>35 weeks gestation) that appear visibly jaundice.^7^ Clinically significant hyperbilirubinemia depends on both the serum bilirubin levels and an infant’s clinical risk factors (e.g., GA and the infant’s age in hours) at the time a serum bilirubin test is completed. This clinical assessment is assisted by using tools like the risk curve nomogram (Bhutani Nomogram), treatment curves and clinical guidelines.^4,7,9^

CPS recommends phototherapy to be initiated as the first-line treatment for infants with hyperbilirubinemia which in turn can pose an increased risk for encephalopathy.^7^ Exchange transfusion (ET) is administered if bilirubin levels continue to rise despite phototherapy use, as it results in a rapid reduction in bilirubin levels.^7,10^ ET is an invasive procedure that has been used for a long time and is considered safe for infants at higher risk for bilirubin encephalopathy but can also be associated with adverse effects like pulmonary hemorrhage or necrotizing enterocolitis.^7,10–12^

Intravenous immunoglobulin G (IVIG) therapy was introduced to treat infants who have hyperbilirubinemia with hemolytic disease (i.e., ABO blood group incompatibility) within the past 20 years, but reports on its effectiveness have been inconclusive.^13,14^ Some studies have reported that IVIG use reduces the need for ET by slowing the rate of hemolysis in addition to reducing the number of phototherapy sessions required.^13,15–18^ Other studies have reported the use of IVIG therapy (a blood product) to treat NHb, could have side effects like pulmonary emboli, anaphylaxis, hypersensitivity, aseptic meningitis and thrombosis.^14^ Consequently, there remains a debate in the literature surrounding the routine use of IVIG therapy to treat NHb, especially those who have a positive direct antiglobulin test (DAT).^13,14^ Nevertheless, the incidence of IVIG usage for hyperbilirubinemia is unknown and Ontario has suffered a shortfall in IVIG supplies since 2019.^19^ Yet, population-level Ontario data have not been utilized to study NHb trends or its treatments.

The objective of this study is to report the current rate of NHb in Ontario (GA ≥35 weeks) and its treatments: phototherapy, IVIG, and ET. Comparing these treatments with data from a large population may help us understand IVIG use and how common NHb is in Ontario. Furthermore, understanding of NHb’s early care may reduce the number of newborns getting such aggressive therapies in the future and reduce potential adverse effects.^20^

## Method

A population-based retrospective cohort study of term and near-term infants (≥35 weeks’ gestation) born in Ontario from April 1, 2014, to March 31, 2018, was conducted. Institutional research ethics board approval was received from St. Michael’s Hospital to access national and provincial databases. For the purposes of this study, infants were deemed to have clinically significant hyperbilirubinemia if the care provider indicated it in the clinical database. The diagnosis was not based on a serum bilirubin threshold as the threshold changes based on clinical factors with each individual infant and is based on a clinical decision.

### Data sources

To determine the incidence of NHb and its management amongst term and near-term infants, provincial and national databases including Better Outcomes Registry & Network (BORN) Ontario and Canadian Neonatal Network (CNN) were accessed.^21–24^ BORN collects data on all live births in Ontario at their institution of birth and CNN collects data on all the infants admitted to Level III neonatal intensive care units (NICU) nationally. These databases have been in existence for more than 10 years and each database follows specific guidelines for data collection.^25–27^ In addition, these databases have been used in numerous peer-reviewed publications.^28–31^ To facilitate estimates regarding the incidence of ETs in level III NICUs, birth rates at level III NICU sites in Ontario were collected from the Provincial Council for Maternal and Child Health.^32,33^

For the purposes of this study, we only used CNN for Ontario Level III NICUs to assess ETs since level III centers are the only place where ETs are performed. The remaining data were collected from BORN. The BORN database was used as it is reported to have high validity of core values (i.e., GA) in terms of its agreement with the patient’s health record and therefore is a good reflection of the neonatal population of interest.^24^ BORN was introduced in 2009 and data from 2014 onwards was utilized.^24^ Within BORN, Ontario birth rate, the proportion of infants who presented with clinically significant hyperbilirubinemia (as per diagnosed by the care provider), the number of infants that underwent treatment for hyperbilirubinemia (phototherapy, IVIG), and the number of infants who had other health conditions alongside NHb were collected. BORN was also used to collect information on the number of infants admitted to the level II or level III NICUs. To avoid duplicate counting, only the CNN database was used to estimate the rate of ET use per year in Ontario. When there were incomplete site-specific data for incidence rates in ET rates in level III NICUs, birth rates at sites in Ontario were collected from the Provincial Council for Maternal and Child Health.^32,33^

The mean maternal age of mothers who gave birth between April 2014 to March 2016 was retrieved from Statistics Canada.^34^

### Data analysis

Descriptive statistics was used to report the incidence of clinically significant NHb and the rate of phototherapy, IVIG and ET use per year and in total. Statistics Canada was used to collect birth rates in Ontario from 2014 to 2018 to estimate the prevalence of clinically significant NHb in Ontario.^34,35^ To estimate the rates of ETs administered in level III NICUs, for years that had incomplete data, the rates were derived from known rates of these treatments reported in CNN. Since ETs are only performed in level III NICUs, the estimated rates do not include duplicates.

Statistical analysis (i.e., hypothesis testing, and tests for trend in proportions) was conducted using MedCALC version bv,^21^ SPSS version 26 and RStudio version 1.3.1073, with a significant *P* value set to the standard value of *P* < 0.05.

## Results

### Incidence of neonatal hyperbilirubinemia in Ontario

**Fig. 1:**
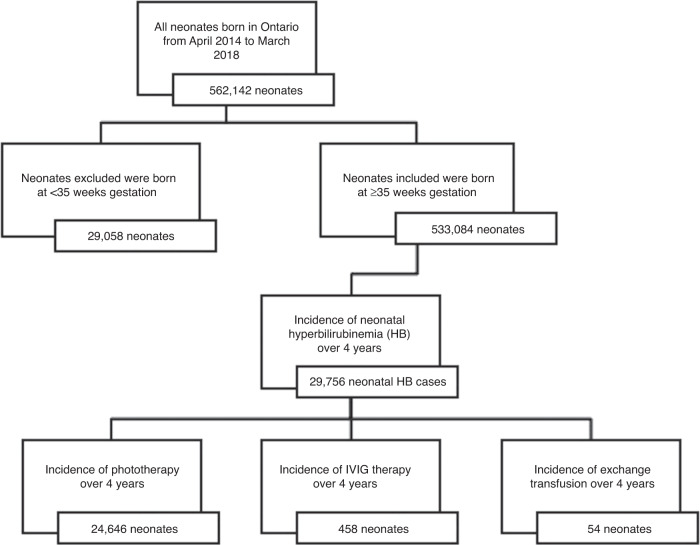
Total number of neonates analyzed. Flow diagram of neonates included in the final analysis basd on the data obtained from BORN, CNN and Statistics Canada.

From April 1, 2014, to March 31, 2018, the mean age of mothers who gave birth was 32 and a total of 562,142 infants were born in Ontario (Table 1).^34,35^ Of all the infants born in Ontario over the 4 years, 288,521 (51.3%) infants were male and 273,621 (48.7%) infants were female.^35^ Of the total infants born in Ontario, 533,084 (94.8%) were born at a GA of ≥35 weeks’ (Fig. 1). A total of 29,756 (5.6%) of these term and near-term infants (≥35 weeks GA) were diagnosed with hyperbilirubinemia. The incidence of clinically significant NHb reported in term and near-term infants over this study period ranged between 5.0 and 5.9% (Table 1). The incidence of clinically significant NHb statistically increased over 4 years (*P* < 0.01). Among the 29,756 infants who developed clinically significant hyperbilirubinemia over the 4 years, 24,646 (82.8%) infants received phototherapy, 54 (0.2%) infants received an ET and 458 (1.5%) infants received IVIG.

**Table 1:** Birth rate, hospital admissions and the incidence of neonatal hyperbilirubinemia in Ontario from April 2014 to March 2018.

	2014–2015	2015–2016	2016–2017	2017–2018	Total
Ontario infant births	139,963	139,924	140,298	141,957	562,142
Males born in Ontario	71,759	71,798	72,103	72,861	288,521
Females born in Ontario	68,204	68,126	68,195	69,096	273,621
Ontario Infants GA ≥35 weeks at birth	133,225	133,222	133,559	133,078	533,084
Level II NICU (neonatal intensive care unit) admissions in Ontario	11,718	11,872	11,792	12,020	47,402
Level III NICU (neonatal intensive care unit) admissions in Ontario	1230	1032	859	1011	4132
Incidence of neonatal hyperbilirubinemia	6712 (5.04%)	7573 (5.68%)	7810 (5.85%)	7661 (5.76%)	29,756 (5.58%)

### Treatment and management of infants with hyperbilirubinemia born at a GA ≥35 weeks

**Fig. 2:**
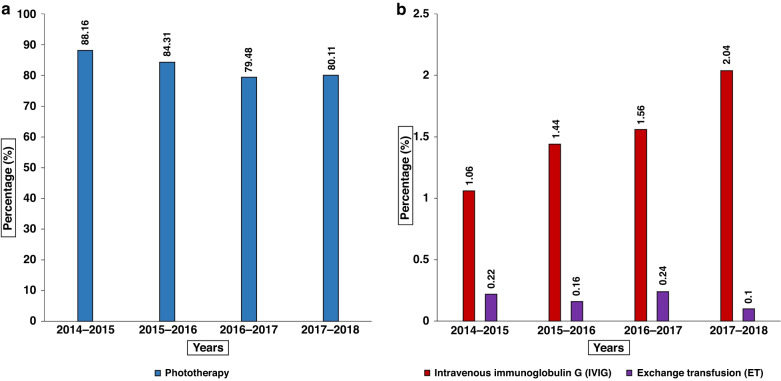
Treatments for hyperbilirubinemia used over time. **a** Bar chart representing the proportion of phototherapy use from April 2014 to March 2018. **b** Bar chart representing the proportion of exchange transfusion (ET) and Intravenous Immunoglobulin G (IVIG) therapy use from April 2014 to March 2018.

Infants with NHb, received phototherapy at a rate between 79.5 and 88.2% during the study. Similarly, the annual rates of IVIG use were between 1.1 and 2.0%, and the annual rates of ET use were between 0.1 and 0.2% (Fig. 2). Although from 2014 to 2018, there was a significant (*P* < 0.01) decrease of 8.1% (88.2 vs 80.1%) in phototherapy administration for infants with hyperbilirubinemia. Among infants with hyperbilirubinemia, there was a significant increase in the rate of IVIG use when comparing 2014 and 2018. In 2014, 71/6712 infants (1.1%) received IVIG and in 2018, 156/7661 infants (2.0%) received IVIG (*P* < 0.01). The proportion of infants with hyperbilirubinemia that received IVIG increased over the 4 years (*P* < 0.01) (Fig. 2). Finally, there was a slight decrease (0.2–0.1%) in ETs administered when comparing the rate in 2014 with the rate in 2018 but this difference was not significant (*P* = 0.315). ETs were used less frequently than phototherapy and IVIG overall. However, among infants with hyperbilirubinemia requiring more intensive treatments (i.e., ET or IVIG), 86/6712 infants (1.3%) born in 2014 and 164/7661 infants (2.1%) born in 2018 (*P* < 0.01) received either ET or IVIG.

## Discussion

Despite significant advancements in the management of NHb our data show that infants in Ontario continue to develop hyperbilirubinemia requiring more intensive treatments than phototherapy (i.e., IVIG and/or ET). Since term and near-term infants continue to develop clinically significant hyperbilirubinemia, infants are at increased risk for developing long-term sequelae including ABE and CBE.^7,10,11,14^

As expected, phototherapy was the most frequently used treatment for term and near-term infants. Being diagnosed with NHb requires monitoring. If the bilirubin level continues to rise, the infant may require further treatment. The small changes in the incidence of NHB and the slight decrease in phototherapy are possibly the results of increased surveillance and monitoring.^36^ Despite this, there was an increase in both the incidence of hyperbilirubinemia and the use of aggressive treatments (i.e., ET or IVIG). The number of infants with hyperbilirubinemia who received IVIG or ET went from 1.3% in 2014 to 2.1% in 2018 and this effect is due to the increase in IVIG use overtime. The administration of IVIG therapy almost doubled its use in Ontario over the 4 years, from 2014 (1.1%) to 2018 (2.0%). These results are consistent with previous research indicating that IVIG as a treatment for NHb is increasing in its frequency.^37^ To our knowledge, there is no existing Canadian data on IVIG use for treating hyperbilirubinemia. This increase in the frequency of IVIG use could be related to the increased physician awareness of IVIG’s use in treating NHb.

In the past 5 years, there has been a Cochrane review examining the effectiveness of IVIG use among term and near-term infants with hyperbilirubinemia.^13^ This review conducted by Zwiers et al. (2018) found that 7 out of 9 studies reported that IVIG use led to a reduction in ET administrations in treating NHb.^13,15,17,38–42^ However, these 7 studies were considered to have a low level of certainty in their results, as most of them did not indicate their procedure for randomization, and/or lacked an appropriate control group to compare the outcomes of IVIG use.^13,15,17,38–42^ Consequently, Zwiers et al. (2018) could not conclude with certainty the effectiveness of IVIG in treating infants who present with hyperbilirubinemia.^13^

The continued use of ET despite the use of IVIG, as seen in the results of this study, warrants an additional review of IVIG.^13^ Other studies have also reported the ineffectiveness of IVIG use amongst DAT-positive infants with ABO incompatibility and hemolysis.^14,43,44^ A positive DAT for infants with ABO hemolytic disease is four times more likely to develop jaundice in comparison to infants who had a negative DAT.^14,45^ Despite the continued debate surrounding the effectiveness of using IVIG therapy to treat DAT-positive (ABO hemolytic disease) infants with hyperbilirubinemia, IVIG is increasingly being used to treat NHb. Irrespective of the reported neonatal outcomes of using both IVIG and phototherapy to treat infants with hyperbilirubinemia, infants who received IVIG also presented with significantly higher rates of blood transfusion, rebound hyperbilirubinemia and severe anemia than those who only received phototherapy treatment.^46^

This study had some limitations. First, this study relied on provincial and national databases that collected manually entered data from neonatal centers in Ontario using a standard online form. We did not conduct chart reviews, so we are limited by the accuracy of this data. Though this is a limitation, these databases have been used to report trends in the incidence of clinically significant NHb and its treatment because the database is still reflective of the neonatal population of interest. Furthermore, in order to minimize this source of error, two provincial databases were used. Appropriate adjustments were made from both databases to ensure rates were not calculated twice. Secondly, there were some sites with incomplete level III NICU data. In order to address this, the CNN database was used to facilitate estimates of the incidence of ET use in Ontario because they are the only available provincial databases reporting on level III NICUs. Finally, both databases were limited in the data provided. For instance, neither databases collected the duration of hospital stay nor the age of diagnosis, admission, and readmission. Similarly, the pre-treatment or peak bilirubin level during diagnosis/treatment was not recorded; therefore, one can only comment on the treatments used but not the bilirubin threshold at which phototherapy, IVIG or ETs were performed. Also, a consistent definition for the severity of NHb was not used and, so the number of infants with severe NHb could not be identified. Therefore, despite phototherapy being the first-line treatment, it is also unclear why not all infants diagnosed with hyperbilirubinemia received phototherapy. Some potential reasons could include infants’ TSB (or TcB) levels being below treatment thresholds or other reasons around data collection that were beyond our control.^24^ In addition, physicians may have diagnosed significant hyperbilirubinemia that they interpreted was below treatment thresholds and had managed the infants by monitoring serum bilirubin levels and follow-up. These infants may not have reached treatment thresholds in subsequent bilirubin measurements.

## Conclusion

Clinically significant NHb still occurs in Ontario, with an increasing number of infants receiving IVIG therapy despite inconclusive evidence to recommend its use. This highlights the necessity of a future prospective study to better determine the effectiveness of IVIG use in treating NHb, especially given the recent shortage in IVIG supply in Ontario.^13^ Future research should assess these other more aggressive treatments (IVIG and ET) and their effect in reducing serum bilirubin beyond phototherapy.

## Data Availability

Data supporting the results of this study can be requested from CNN and BORN Ontario. Additional supporting data can also be found in Statistics Canada.
